# Chronic stress, epigenetics, and adipose tissue metabolism in the obese state

**DOI:** 10.1186/s12986-020-00513-4

**Published:** 2020-10-19

**Authors:** Yang Xiao, Dongmin Liu, Mark A. Cline, Elizabeth R. Gilbert

**Affiliations:** 1 Department of Animal and Poultry Sciences, Virginia Polytechnic Institute and State University, Blacksburg, VA USA; 2 Department of Human Nutrition, Foods, and Exercise, Virginia Polytechnic Institute and State University, Blacksburg, VA USA; 3 School of Neuroscience, Virginia Polytechnic Institute and State University, Blacksburg, VA USA

**Keywords:** Adipose tissue, Obesity, Stress, Epigenetics, Metabolism, Nutrition

## Abstract

In obesity, endocrine and metabolic perturbations, including those induced by chronic activation of the hypothalamus–pituitary–adrenal axis, are associated with the accumulation of adipose tissue and inflammation. Such changes are attributable to a combination of genetic and epigenetic factors that are influenced by the environment and exacerbated by chronic activation of the hypothalamus–pituitary–adrenal axis. Stress exposure at different life stages can alter adipose tissue metabolism directly through epigenetic modification or indirectly through the manipulation of hypothalamic appetite regulation, and thereby contribute to endocrine changes that further disrupt whole-body energy balance. This review synthesizes current knowledge, with an emphasis on human clinical trials, to describe metabolic changes in adipose tissue and associated endocrine, genetic and epigenetic changes in the obese state. In particular, we discuss epigenetic changes induced by stress exposure and their contribution to appetite and adipocyte dysfunction, which collectively promote the pathogenesis of obesity. Such knowledge is critical for providing future directions of metabolism research and targets for treating metabolic disorders.

## Introduction

Adipose tissue, in addition to providing a reservoir of energy, serves as an endocrine organ, as it synthesizes and secretes a diverse array of hormones and cytokines that regulate metabolic homeostasis and control various physiological functions such as energy metabolism, thermoregulation, food intake, and glucose homeostasis [1–3].

There are two major types of adipose tissue—white adipose tissue (WAT), which is mainly composed of white adipocytes that are designed for energy storage, and brown adipose tissue (BAT), containing brown adipocytes that largely function to dissipate energy in the form of heat during non-shivering thermogenesis [4]. More recently, a third type of adipocyte—beige (also called brite/inducible BAT), was found to arise in WAT in response to various cues such as chronic cold exposure and sympathomimetic treatments, with thermoregulatory and energy balance functions [5].

WAT is distributed throughout the body mainly in the subcutaneous (sWAT), visceral (vWAT), inter- (itMAT) and intramuscular (iMAT) as well as bone marrow (MAT) depots [6], among which sWAT and vWAT are of the most metabolic importance. sWAT emerges prior to vWAT during embryonic development [7]. sWAT is located underneath the skin and is primarily responsible for energy storage [8]. In humans, about 80% of body adipose tissue is sWAT, with the main depots being abdominal, subscapular (upper back), gluteal and thigh, while vWAT contributes to 6–20% of total body fat [9]. vWAT is distributed inside the peritoneum and around internal organs (e.g. stomach, liver, intestines and kidneys), and protects the tissue as physical padding [8]. vWAT can be sub-divided into mesenteric, retroperitoneal and omental depots in humans, and also the perigonadal depot in rodents, in accordance with its location [8]. Increased vWAT accumulation is positively associated with the onset of metabolic diseases [10], whereas accretion of sWAT is more benign, having little association with the development of metabolic disorders [11]. There are two cellular mechanisms for adipose tissue expansion: hypertrophy and hyperplasia. Hypertrophy is characterized by enhanced triglyceride storage and associated expansion of existing adipocytes, while hyperplasia refers to the increase in adipocyte cell number. Hypertrophy is predominant in adulthood obesity as it is observed in all overweight and obese individuals [12], whereas hyperplasia is a mark of the severity of obesity [13].

Stress may play a role in the development of obesity. Cortisol is an essential glucocorticoid involved in the stress response. Adult males with visceral obesity had increased awakening cortisol responses (analyses based on salivary samples), whereas adult females with subcutaneous obesity had no such change [14]. Though no association was found between body mass index (BMI) and urinary cortisol secretion regardless of sex [15], a positive correlation was observed between BMI and urinary cortisol metabolite (5α- and 5β-tetrahydrocortisol) excretion [16]. 11β-hydroxysteroid dehydrogenase type 1 (11β-HSD1), which converts cortisone to cortisol, with its overexpression associated with central obesity, had increased activity in the adipose tissue and reduced hepatic activity in adult females with a higher BMI [16]. Maternal obesity also induced higher baseline cortisol in the offspring, with an impaired stress response [17] and predisposition to metabolic alterations and adiposity [18]. These results collectively indicate that stress exposure is associated with alterations in adipose tissue metabolism and the pathogenesis of obesity.

This review focuses on the relationship between stress and adipose tissue physiology in the obese state. Integrating knowledge from human clinical trials and animal models, we discuss endocrine, genetic, and epigenetic changes in adipose tissue as a consequence of early- and later- life stress exposure. Such knowledge is critical to provide new insights for the direction of research in nutrition and metabolism.

### Fat mass and adipose tissue distribution

World Health Organization (WHO) defines obesity as “abnormal or excessive fat accumulation that may impair health” and a BMI ≥ 30 is classified as obese in adults, whereas for young children and adolescents, the standard was made according to the WHO Growth Reference [19]. Current studies also adopt a percentage body fat ≥ 25% in men and ≥ 35% in women as a criterion for obesity diagnosis [20]. Gender, age and ethnic group are the major variables predicting fat mass in obesity [21]. The US National Health and Nutrition Examination Survey (NHANES) reported higher prevalence of extreme adiposity (on the basis of the 80th percentile of percentage body fat) in Mexican-Americans than non-Hispanic (NH) whites and blacks in boys but not girls aged 8–19 years [22]. In adults, fat mass tended to be higher in white men and women compared to black men and women, respectively, at a fixed BMI [23]. Young adults (18–29 years) had a lower percentage of fat mass than the elderly (≥ 70 years), and there were differences among ethnic groups (Mexican–American > NH white > NH black) whereas there was no difference among these groups in the elders regardless of sex [24]. Moreover, without a significant difference in BMI, middle-aged overweight/obese women had higher percentage body fat masses than men [25]. According to NHANES 1999–2004, percentage extremity fat (of the total mass in the corresponding arm/leg) in males from all ethnic groups decreased during adolescence then increased again throughout later life at the same percentile of the age groups, whereas in women, percentage of extremity fat continued increasing until 79 years of age, then dropped slightly in later life. Moreover, in males among all ethnic groups, percentage extremity fat had a lower reduction during adolescence and greater increase in later life in those in the ≤ 50th percentile than those in the > 50th percentile, and the reduction in percentage leg fat continued until 59 years of age before it went back up in those in the > 50th percentile. In females among all ethnic groups, percentage extremity fat had a greater increase until 79 years of age in those in the ≤ 50th percentile than those in the > 50th percentile, whereas the decrease later in life did not differ among percentiles. In males among all ethnic groups, like the percentage extremity fat, the percentage trunk fat reduces during adolescence and then increases again throughout later life, with a slight reduction after 79 years of age. This reduction during adolescence was higher in those in the > 50th percentile than those in the ≤ 50th percentile in Mexican Americans, but not NH white or black individuals, while the increase later in life was lower in those in the > 50th percentile among all ethnic groups. In females among all ethnic groups, there was a greater increase in percentage trunk fat until 79 years of age in those in the ≤ 50th percentile, whereas a greater reduction from 79 years of age onward was only observed in the ≤ 50th percentile of Mexican Americans [26]. At BMI = 30, Chinese and South Asians (adjusted for age and sex by cohort means) had more vWAT than the Europeans, while sWAT was similar between Chinese and Europeans but greater in South Asians [27].

Brown fat also changes during the progression to obesity. BAT activity is greater in lean healthy females than in males, and in young people than in older adults [28]. In BAT-active subjects, BAT function is inversely correlated with BMI [29] as well as percentage body fat mass, whereas those with BMI > 35 and percentage body fat mass > 30% barely showed any detectable BAT activity [30]. Specifically, obese male subjects had a lower percentage of active BAT volume in all 6 depots (cervical, supraclavicular, axillary, mediastinal, paraspinal and abdominal) and lower BAT activity in 5 depots (except cervical) than their lean healthy counterparts. The greatest difference in the volume was observed in the paraspinal depot, while the activity difference differed the most in the supraclavicular depot—the most BAT-active depot in healthy lean males, between lean and obese males [31]. The main findings of these studies are summarized in Table 1.

**Table 1:** Fat mass and adipose tissue distribution

Reference	Gender	Age	Race	BMI	Main findings
[22]	Both	8–19	Mexican American Non-Hispanic black Non-Hispanic white	Boys^a^: 21.8 ± 0.14 Girls: 22.1 ± 0.15	Overall prevalence of adiposity is positively associated with BMI in children and adolescents Prevalence of high BMI (≥ 95 percentile of BMI-for-age) and high adiposity (≥ 80th percentile of percentage body fat) are higher in Mexican–American than non-Hispanics boys, whereas high BMI has higher prevalence in non-Hispanic black girls Prevalence of high adiposity in all BMI groups are lowest in non-Hispanic black boys and girls Body fat (%) is lower in non-Hispanic blacks than Mexican-Americans, but BMI is lower in non-Hispanic black boys while higher in girls than Mexican–American counterparts
[23]	Both	≥ 20	Hispanic Mexican American Non-Hispanic black Non-Hispanic white	≥ 25	No overall difference in the prevalence of obesity between males and females Non-Hispanic black women have the highest prevalence of obesity than women in other ethnic groups Prevalence of severe obesity (BMI > 35) is higher in non-Hispanic blacks than whites in both sexes Whites have relatively higher fat mass than blacks at a fixed BMI
[25]	Both	53.5 ± 13.9	Caucasian descent	27.8 ± 4.9	In both males and females, those without metabolic syndrome have lower BMI, body fat (%), waist circumferences, waist to hip ratios, fasting plasma insulin and glucose levels, plasma triglyceride and uric acid levels, as well as systolic blood pressure together with higher HDL than those with metabolic syndrome Body fat (%) is positively correlated with age, BMI, waist circumference and waist to hip ratio, while negatively correlated with height Overall body fat (%) is higher in overweight/obese females than males, whereas waist circumference is higher in age-matched males
[26]	Both	≥ 8	Mexican American Non-Hispanic black Non-Hispanic white	All ranges^b^	Females have higher absolute and relative fat mass than males at all ages Trunk and extremity fat (%) are lower in males than females at all ages in all ethnic groups, and lower in adolescence then increased in adulthood in males whereas both increased to 79 years of age in females Extremity fat (%) reduction is lower and increase is higher in males in the ≤ 50 th percentile than > 50 th percentile from all ethnic groups, whereas for trunk fat (%), this is only in Mexican-Americans Trunk and extremity fats (%) have higher increase in females in the ≤ 50 th percentile than > 50 th percentile from all ethnic groups
[27]	Both	30–65	Aboriginal Chinese European South Asian (living in Canada)	≥ 18.5	Body fat (%) is higher in Aboriginals and South Asians than Europeans vWAT and sWAT are lower in Europeans than in Chinese and South Asians
[28]	Both	Middle aged^c^	N/A	Not specified^c^	Females have greater amount and more active BAT than males BAT activity is greater in young than older adults and in people with lower BMI and fasting glucose levels
[29]	Both	Male: 35.8 ± 9.0 Female: 38.8 ± 8.8	N/A	Male: 23.8 ± 2.6 Female: 21.1 ± 2.3	Under cold exposure, younger subjects have higher BAT activity than older ones, and BAT activity is mainly found in supraclavicular and paraspinal regions BAT activity is inversely related to BMI, vWAT and total fat mass
[30]	Both	39.2 ± 8.1	N/A	42.1 ± 3.8	Obese individuals have reduced cold-induced thermogenesis and BAT activity
[31]	Male	Lean: 22.5 ± 4.9 Obese: 28.8 ± 4.7	N/A	Lean: 23.2 ± 1.9 Obese: 34.8 ± 3.3	Obese males have less BAT activity than lean males, but more potential to achieve BAT expansion upon cold exposure BAT is highly concentrated in cervical, supraclavicular, axillary, mediastinal, paraspinal, and abdominal regions

^a^BMI was calculated as [body weight (kg)/height^2^ (m^2^)] as in adults, but adiposity was described as “percentiles of BMI-for-age” from the CDC growth charts

^b^Distribution of BMI and the prevalence of obesity was determined within each age group

^c^Average age and BMI were calculated within BAT positive versus BAT negative groups, not given for the sampling population

### Depot-specific adipose tissue physiology

Obese females store more dietary fat in sWAT, especially in the lower body, whereas vWAT is the preferred depot for dietary fat storage in obese males [32]. A recent study identified 414 differentially-expressed genes between sWAT and vWAT, among which 60 were associated with obesity-related traits (e.g. waist to hip ratio) [33]. Pathway analysis revealed that genes involved in extracellular matrix (ECM) remodeling were highly enriched in the sWAT, which may confer the functional morphology of adipocytes in sWAT to exert a protective effect, while genes involved in inflammation were highly enriched in vWAT, which confirms its role in the pathogenesis of metabolic diseases. Moreover, developmental genes belonging to the homeobox (HOX) family were also differentially expressed in sWAT and vWAT, with HOXC and HOXD clusters being highly expressed in sWAT and HOXB being highly expressed in vWAT, which may contribute to the functional characteristics of the depots [33]. Genes related to adipocyte metabolism (proliferation, differentiation, lipid turnover, etc.), adipokine secretion, and ion channels and cell signaling are also differentially expressed in sWAT and vWAT, according to transcriptome studies [34–36].

Upon exposure to excessive lipids in the circulation, hypertrophy occurs to adapt to extra energy storage, which increases fat mass and triggers hyperplasia [37]. Preadipocytes from sWAT express more of adipogenic master regulators peroxisome proliferator-activated receptor gamma (PPARγ) and CCAAT/enhancer binding protein α, and thus these cells are more capable of extensive replication and rapid adipogenesis than the preadipocytes from vWAT. Interestingly, the vWAT preadipocytes show a catch-up growth pattern after 60 days of culturing in vitro [32]. Additionally, an increase in endocannabinoids, which promote adipogenesis and triglyceride uptake through the upregulation of PPARγ, was only observed in visceral obesity [38]. Postprandial free fatty acid (FFA) release is greater in abdominal than in lower body obesity, indicating a faster rate of lipolysis in the visceral adipocytes which are inherently resistant to the antilipolytic effect of insulin [39]. Moreover, vWAT is more susceptible to TNFα-induced apoptosis, whereas sWAT is more sensitive to aging and sequential loss of lipid storage capacity (reviewed in [32]).

Expansion of adipose tissue in the obese state has been categorized into two types: metabolically healthy—with preferred lipid storage in sWAT, and metabolically unhealthy—with excessive vWAT and ectopic fat accumulation [40]. Accumulation of sWAT in the lower body is associated with lower circulating triglycerides and glucose but greater high density lipoprotein (HDL) and insulin sensitivity, while accumulation of abdominal sWAT may be involved in the pathogenesis of insulin resistance and cardiovascular diseases, although the association is not as strong as for vWAT [38]. Impaired expandability of sWAT upon excessive lipid storage can result in the accumulation of vWAT [40]. As vWAT attaches and shares the vasculature with internal organs, lipid metabolites and adipokines are directly secreted from these adipocytes into the portal vein, leading to the disruption of homeostasis in hepatocytes and impacts on glucose and lipid metabolism, which eventually contribute to the onset of insulin resistance [38]. Apart from sWAT and vWAT, in contrast to the increased MAT that is accompanied by lowered mineral density in anorexia nervosa, obese adolescents [41] and adults (excluding postmenopausal women) [42] had less MAT with greater bone mineral density than healthy controls, while postmenopausal obese women had increased MAT due to the lack of estrogen [43] but the association between MAT and total fat/vWAT/sWAT masses were controversial among studies, which may suggest a unique role of MAT in endocrine modulation and the progression of adiposity [42]. To the contrary, both itMAT and iMAT increased in obese subjects, but only itMAT had a similar distribution and association with inflammation and insulin resistance as in vWAT [6].

Rapid expansion of adipose tissue with limited vascularization induces hypoxia in obesity. The hypoxic adipocytes secrete chemokines which attract macrophages and trigger the inflammatory responses [44]. Many proinflammatory cytokines, including but not limited to those from the interleukin cytokine family, interferon γ, TNFα, monocyte chemoattractant protein 1, and plasminogen activator inhibitor 1, etc., are more abundant in vWAT than in sWAT, which may be related to the enriched lymph nodes and milky spots for immunocompetent molecules in visceral depots such as the omentum [32, 38]. Hypertrophy of adipocytes from vWAT in the obese state stimulates the secretion of proinflammatory cytokines in the adipose tissue and results in increased vascular permeability, which attracts macrophages. Increased infiltration and proliferation of macrophages in adipose tissue sustains the inflammatory response, and further stimulates lipolysis in the surrounding adipocytes to provide energy for the immune system. The increased circulating FFAs in turn facilitate the maturation of preadipocytes and adipocyte hypertrophy, which ultimately leads to metabolic dysregulation [45, 46].

Interestingly, BAT also recruits macrophages and secretes proinflammatory cytokines, which trigger the inflammatory response. Inflammation in BAT inhibits the proliferation of brown adipocytes through interrupting catecholamine signaling and the differentiation of brown adipocytes by downregulating PPARγ, while promoting apoptosis of brown adipocytes through TNFα, which together alter the thermogenic activity of BAT [47, 48].

In summary, in response to increased energy intake, adipose tissue expands in a depot-dependent manner, with differences between white and brown tissue activity. These changes likely determine whether the onset of obesity will be associated with metabolic disruptions that have negative health consequences such as the case with preferential accumulation of vWAT. Obesity can be described as a chronic state of mild inflammation, and adipocyte hypoxia and macrophage infiltration likely lead to changes in circulating cytokines and hormones that influence metabolism and other physiological processes, thereby exacerbating the pathogenesis of the obese condition.

### Adipokines, lipokines and endocrine disruption in obesity

Adipose tissue is one of the largest endocrine organs, secreting a diverse assortment of hormones and pro-inflammatory factors [2]. In addition, both white and brown adipocytes secret exosomes, which is the major source of circulating microRNAs (miRNAs) [49]. These miRNA-containing exosomes are sequentially taken up by cells in other tissues. Therefore, exosomal miRNAs are considered to be another type of adipokine, and essential for intra- and inter-tissue cell-to-cell communications to regulate energy homeostasis [50]. The abundance of vWAT exosomes were found to be negatively correlated with BMI in teenage girls [50]. Among those differentially expressed miRNAs in vWAT between obese and lean girls, the majority are involved in TGF-β and Wnt/β-catenin signaling pathways, which regulate adipogenesis and inflammatory responses [51, 52]. In morbidly obese men, plasma miR-130b, which promotes adipose tissue inflammation and insulin resistance [53], and miR-423-5p, which promotes angiogenesis [54], were decreased [55]. Interestingly, both were increased in obese prepubertal children [56], suggesting age and severity differences in circulating miRNA levels in obesity.

In BAT, expression of exosomal miR-92a was upregulated and accompanied by whitening of the BAT in mice fed a high fat diet (HFD). Meanwhile, serum exosomal miR-92a level was negatively correlated with BAT activity and BAT glucose uptake rates in healthy adults [57]. miR-92a was later found to be highly expressed in the endothelial cells of the vascular wall in patients with advanced coronary atherosclerotic plaques. Its target genes are actively involved in inflammation and atherosclerosis [58]. These results suggest that miR-92a may be used as a marker to track BAT activity and monitor inflammatory responses during the early onset of obesity.

Lipids secreted by adipose tissue that function as signaling molecules among tissues to regulate lipid metabolism are called lipokines [59]. Endogenous lipokine 12,13-dihydroxy-9Z-octadecenoic acid (12,13-diHOME) concentration was positively correlated with BAT activity upon cold exposure and negatively correlated with BMI, circulating triglycerides, leptin and fasting plasma insulin and glucose levels [60]. It enhanced fatty acid uptake and lipid oxidation in BAT activated by β3-adrenergic stimulation [60]. However, in diet-induced obese mice, exogenous supplementation of 12,13-diHOME for 2 weeks did not alter body weight, circulating FFAs or glucose tolerance albeit there was a decrease in circulating triglycerides [60]. Further research is required to fully elucidate its physiological effects.

Another type of adipose-derived lipokine is fatty acid esters of hydroxy fatty acids (FAHFAs), which are composed of one fatty acid molecule and one hydroxy fatty acid molecule on an ester bond [61]. A total of 51 families of FAHFA exist in both WAT and BAT [62]. The major effects of FAHFAs include reducing inflammation mediated by adipose-derived macrophages, increasing glucose-induced insulin secretion and insulin-stimulated glucose uptake in adipocytes by enhancing glucose transporter type 4 (GLUT4) translocation [63]. Thus, FAHFAs have been proposed as a promising treatment for type II diabetes. Although individuals with insulin resistance had reduced FAHFA levels in sWAT [63], little is known about the function of FAHFAs in adipose tissue metabolism, for instance, effects on lipid uptake and circulating lipid profiles. To date, no association of FAHFA abundance and fat mass has been reported.

Leptin is an adipose-derived cytokine (adipokine) that is secreted in proportion to body fat mass [64]. Although rapid accumulation of adipose tissue in the obese state is accompanied by elevated leptin, which regulates energy homeostasis by suppressing food intake and promoting energy expenditure, decreased amounts of soluble leptin receptors as well as reduced leptin transport across the blood brain barrier (BBB), and reduced leptin signaling in the hypothalamus result in hyperleptinemia and eventually leptin resistance [65]. Moreover, there is growing evidence that leptin resistance can be partly attributed to obesity-associated hypothalamic inflammation [66]. The hypothalamic arcuate nucleus (ARC), which contains first-order appetite regulatory neurons neuropeptide Y (NPY)/agouti-related peptide (AgRP) and proopiomelanocortin (POMC)/cocaine-amphetamine-regulated transcript (CART) [67, 68], is a primary site of leptin action. Leptin suppresses food intake by upregulating *POMC* mRNA while inhibiting *NPY*/*AgRP* mRNA in the ARC [69]. Reduced leptin transport across the BBB leads to decreased production of POMC, which suppresses food intake [70]. Mice with the leptin receptor gene knockout in brain endothelial and epithelial cells consumed more of a high-fat, but not chow diet, and had more body fat, indicating a higher sensitivity to food reward under the obese state due to reduced leptin receptor expression (71).

In addition to suppressing food intake, leptin also promotes lipolysis and fatty acid oxidation in adipose tissue as well as insulin-stimulated glucose uptake and oxidation in muscle while inhibiting hepatic gluconeogenesis and insulin synthesis and secretion in the pancreas [72]. Leptin resistance thus results in decreased lipolysis, fatty acid oxidation, and insulin sensitivity but increased hepatic glucose production and circulating insulin levels. Increased circulating glucose and insulin together with decreased insulin sensitivity contribute to hyperglycemia and hyperinsulinemia, while excessive circulating lipids resulting from decreased fatty acid oxidation lead to hyperlipidemia [70]. Detailed molecular mechanisms of leptin regulation of glucose and lipid metabolism are reviewed in [73].

While an increase in fat mass influences central control of energy intake, disruption of energy intake further induces abnormalities in peripheral lipid metabolism and glucose homeostasis. In the obese state, circulating ghrelin—an orexigenic factor secreted in the stomach and ARC, is reduced due to excessive energy intake [74]. Ghrelin promotes food intake through the activation of NPY/AgRp neurons in the ARC, which is also mediated by uncoupling protein 2 (UCP2) [75]. UCP2 is highly expressed in adipose tissue and contributes to the regulation of ATP production, mitochondrial function, and lipid metabolism. Its expression is reduced in obese subjects, with more of a reduction in the abdominal vWAT than sWAT compared to lean healthy controls, regardless of an overall greater expression in vWAT than sWAT [76]. The sWAT UCP2 is positively correlated with both circulating and depot-specific adiponectin levels, and lowered adiponectin is associated with insulin resistance and dyslipidemia in obese subjects [76]. In contrast, expression of UCP2 in pancreatic islets was higher in obese than lean mice, which further led to reduced ATP production and decreased secretion of insulin [77]. Increased β-cell mass and insulin secretion were observed in diet- and gene mutation-induced obese mice, which may compensate for the increased metabolic demand because of insulin resistance under the obese state [78]. However, insulin resistance will gradually progress to overt diabetes when islets fail to compensate for insulin resistance due to β-cell dysfunction and apoptosis as the expansion of mass gradually decreases, which is coupled with increased cellular apoptosis and dysfunction which could contribute to reduced insulin sensitivity and eventually insulin resistance (detailed mechanisms reviewed in [79]).

As the endogenous ligand of the growth hormone (GH) secretagogue receptor (GHS-R), reduced ghrelin is associated with lowered GH levels in obesity [80]. Decreased GH release from the anterior pituitary to the circulation and faster GH clearance may contribute to declined synthesis of insulin-like growth factor 1 (IGF-1) in adipose tissue (although circulating IGF-1 tends to be normal in most studies), which together are associated with reduced lipolysis and the accumulation of fat, especially in vWAT [81].

Leptin and ghrelin not only affect food intake and glucose and lipid metabolism, but also reproductive physiology in obesity. While ghrelin decreases firing activity of gonadotropin-releasing hormone (GnRH) neurons, and suppresses secretion of luteinizing hormone (LH) in humans and rodents [82], kisspeptin, which is regulated by leptin [83], promotes sex hormone secretion via modulating GnRH pulse [84]. Decreased expression of kisspeptin in the ARC was observed in female mice that are centrally resistant to leptin signaling and prone to obesity-induced infertility [85]. Similarly, in HFD-induced male mice, increased circulating leptin and decreased testosterone and LH were observed together with decreased expression of leptin receptor, kisspeptin, and GnRH, indicating a role in obesity-induced male hypogonadism [86]. Consistent with this, lower LH and follicle-stimulating hormone (FSH) levels and lower sex hormone binding globulin (SHBG) were observed in both premenopausal [87] and postmenopausal [88] obese females, and lower total testosterone, FSH, and SHBG were observed in obese males compared to their respective healthy counterparts [89]. However, total estrogen was lower in premenopausal but higher in postmenopausal obese females compared to respective age-matched healthy counterparts [90] with no difference in free estrogen regardless of the menopausal state [91]. Both free and total testosterone levels increased in obese females regardless of the menopause state compared to the healthy counterparts, with an overall higher testosterone level in premenopausal females [91]. In contrast, there was more free estrogen in obese than healthy males [89]. In males, lowered hepatic production of SHBG due to the disruption of endocrine homeostasis under the obese state, such as hyperinsulinemia, resulted in lowered binding of testosterone and thus more free testosterone. As adipose tissue expansion promotes the production of aromatase, an enzyme that converts free testosterone to estrogen, low total testosterone is often observed in obese males, which alters the negative feedback from GnRH and further induces hypogonadism [92]. In the healthy state, testosterone stimulates catecholamine-induced lipolysis and inhibits triglyceride uptake in abdominal adipose tissue. Thus, reduced testosterone in obese males contributes to increased vWAT accumulation to further exacerbate obesity, forming a “self-perpetuating cycle” [93].

Intriguingly, in contrast to the lowered testosterone in obese males, in obese females, hyperinsulinemia promotes the ovarian production of androgens, leading to increases in both total and free testosterone, or hyperandrogenism [92]. Estrogen production in postmenopausal females is solely dependent on the conversion of androgen by aromatase, whereas premenopausal females can also produce estrogen in functional ovaries and the metabolic clearance rate is higher than in postmenopausal females [94]. Therefore, increased estrogen in postmenopausal obese females may be due to the conversion of androgen while decreased levels in premenopausal obese females may be attributed to simultaneously increased clearance of estrogen [90]. An increased clearance of estrogen in premenopausal obese females may be protective for ovary functions as hyperestrogenism could induce endometrial hyperplasia and thereby impair fertility [92]. Estrogen upregulates the expression of antilipolytic α2A-adrenergic receptor only in sWAT [95]. Meanwhile, the estrogen receptor is highly expressed in sWAT in females [96]. Thus, premenopausal females preferentially deposit fat in sWAT. Due to the dramatic reduction in endogenous estrogen and increase in androgens, fat accumulation shifted from sWAT to vWAT in postmenopausal females, leading to the increased propensity for metabolically unhealthy obesity [96]. Moreover, estrogen suppresses appetite through the direct stimulation of central POMC/CART neurons and inhibition of NPY/AgRP neurons in the ARC, as well as potentiating the release of satiating cholecystokinin peptide from the small intestine upon food consumption. Testosterone, in contrast, increases food consumption frequency through the suppression of POMC/CART [97]. Thus, fluctuation of sex hormones in the obese state may exacerbate fat mass accumulation and endocrine disturbances through the promotion of food intake.

Obesity is also directly associated with an altered stress response. Hyperactivation of the hypothalamus–pituitary–adrenal (HPA) axis is observed in obesity. There is greater production of corticotropin-releasing factor (CRF) from the hypothalamic paraventricular nucleus (PVN) that initiates the stress response and the pituitary’s release of adrenocorticotropic hormone (ACTH), which sequentially promotes the secretion of glucocorticoids [98], in obese than lean subjects [81]. Meanwhile, increased urinary-free cortisol was only observed in abdominal obesity [81]. This may be explained by the fact that vWAT has more abundant mineralocorticoid receptors (MR), which have higher cortisol binding affinity than glucocorticoid receptor (GR), compared to sWAT, and that the expression of MR in human fat, especially in vWAT, is positively related to BMI [65]. Elevation of cortisol in obesity results from the expansion of adipose tissue mass, where the highly expressed enzyme 11β-HSD1 converts the inactive cortisone to active cortisol [92]. Cortisol promotes hepatic gluconeogenesis whereby insulin secretion is stimulated, and lipoprotein lipase (LPL) activity is enhanced in abdominal adipose tissue to further exacerbate vWAT accumulation [81]. In addition, both insulin and glucocorticoids regulate NPY expression in vWAT, which was higher in centrally obese rats [99]. In turn, NPY promotes adipocyte proliferation and differentiation, and long-term NPY overexpression induces adipose tissue insulin resistance [100]. Aside from NPY, the HPA axis and orexin interactively stimulate each other under stress exposure to regulate adipose tissue and insulin homeostasis. Central infusion of orexin A activates CRF neurons and downstream effectors [101]. Exogenous orexin A also directly stimulates cortisol secretion from human adrenocortical cells [102]. Meanwhile, central CRF administration also activates orexin neurons [103]. Central activation of orexin stimulates food intake [104], which may contribute to sequential accretion of fat. In HFD-induced obese rats, expression of orexin receptor 1 (OX1R) was decreased in vWAT and negatively correlated with fat mass, plasma triglycerides and fasting insulin [105]. Orexin A administration increased *GLUT4* mRNA and triglyceride content in differentiated 3T3-L1 adipocytes via OX1R [105]. Exogenous orexin A also decreased blood glucose and increased insulin and leptin levels in mice [106]. These results collectively suggest a therapeutic potential of orexin A/OX1R in stress-induced metabolic abnormalities.

**Fig. 1:**
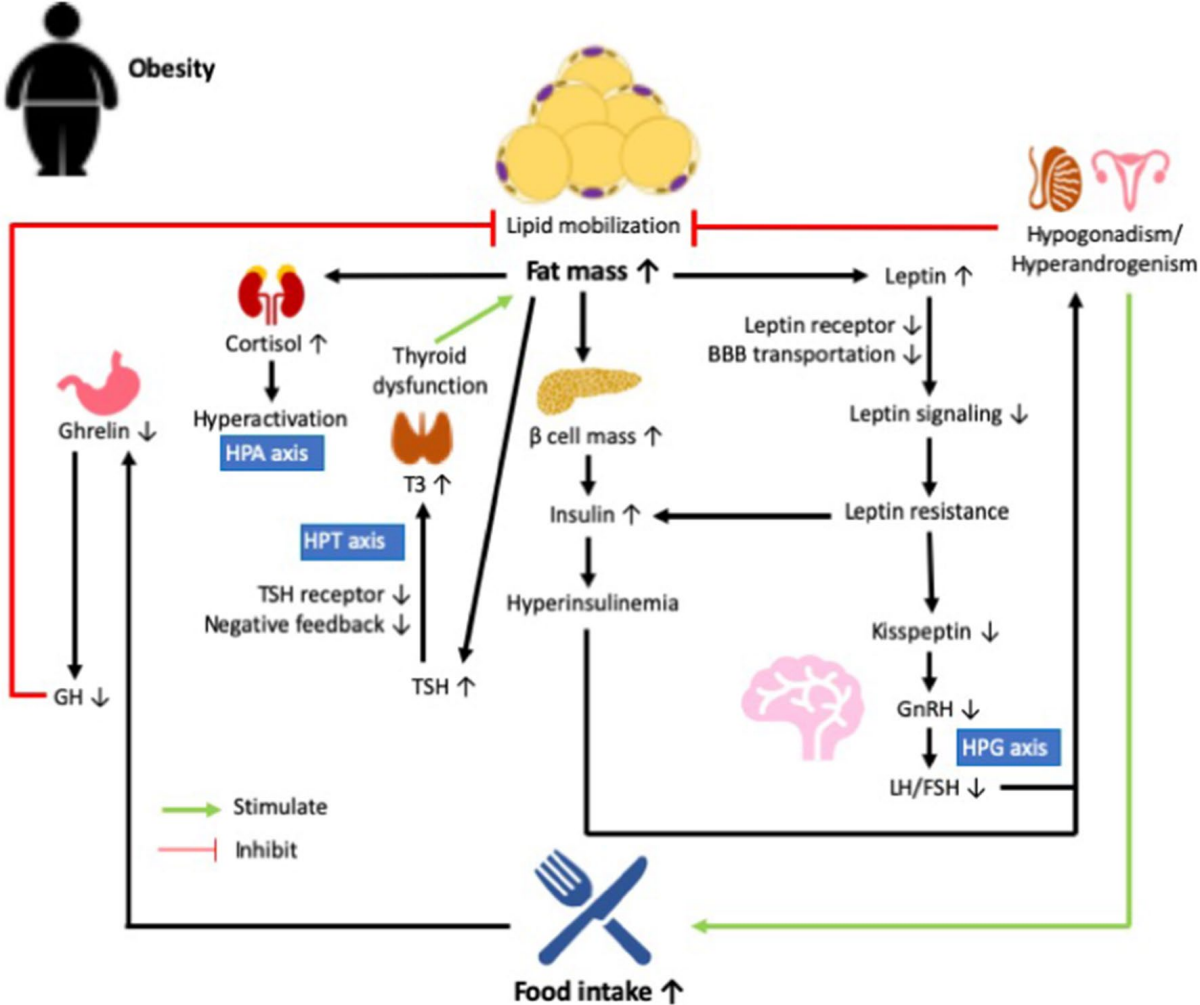
Endocrine disruptions in the obese state. Increased fat mass in obesity results in greater secretion of leptin from adipose tissue, while the expression of leptin receptor and transport of leptin across the blood–brain barrier (BBB) are reduced, leading to lowered leptin signaling and eventually leptin resistance. Sequentially, kisspeptin, which is modulated by leptin, is lowered, leading to hypogonadism in obese males and hyperandrogenism in obese females through the regulation of the hypothalamic-pituitary–gonadal (HPG) axis. Meanwhile, expansion of β-cell mass in the obese state is associated with increased production of insulin, which is further exacerbated by leptin resistance, leading to hyperinsulinemia. Hyperinsulinemia also contributes to the disturbance in sex hormones in obese males and females, which eventually promotes food intake while inhibiting lipid mobilization. Increased food intake inhibits the secretion of ghrelin, resulting in decreased production of growth hormone (GH) and reduced lipid mobilization. Moreover, an increase in fat mass in the obese state promotes the release of thyroid-stimulating hormone (TSH), which then increases the activation of triiodothyronine (T3) via the hypothalamic-pituitary-thyroid (HPT) axis, while the expression of TSH receptor is lowered in the obese state, leading to reduced negative feedback and more release of free TSH and T3 into the circulation. These events lead to thyroid dysfunction and reduced energy expenditure which eventually contribute to fat mass accretion. Finally, obesity is associated with increased production of cortisol and hyperactivation of the hypothalamic–pituitary–adrenal (HPA) axis, which abnormally modulates stress responses later on, thereby influencing food intake and in turn fat mass

Alterations in the HPA axis in the obese state may also lead to thyroid dysfunction. The release of thyroid-stimulating hormone (TSH) from the anterior pituitary is inhibited by elevated cortisol [107], whereas circulating TSH is positively correlated with BMI and leptin, although obese subjects had a similar thyroid hormone profile as healthy counterparts [108]. Free triiodothyronine (T3) and total T3 levels in obese subjects were further positively correlated with TSH [109]. The slight increase in TSH and T3 may result from enhanced gene expression in the PVN guided by leptin [110]. Increased T3 was postulated to be an adaptation to the positive energy balance in the obese state as T3 promotes energy expenditure [111]. However, lower expression of TSH and T3 receptors were found in the adipose tissue of obese patients, leading to more free TSH and T3 but less negative feedback, which further stimulates the production of TSH and T3, eventually leading to thyroid hormone resistance and thyroid dysfunction [110] (summarized in Fig. 1).

In summary, accretion of excessive adipose tissue induces changes in the endocrine system, which results in altered appetite, reproduction and stress responses. Such changes in turn negatively affect lipid metabolism and adipocyte function, eventually exacerbating the obese and inflammatory state and may induce other chronic metabolic diseases.

### Genetic association of obesity and lipid metabolism

Endocrine changes in obesity are associated with differences in gene expression regulation, which can be affected by genetic background or epigenetic modifications. There are two types of genetic obesity—monogenic, which results from a single gene mutation with or without syndromes like cognitive defects, and polygenic, which is caused by the interplay of multiple genes with moderate interaction with the environment [112].

Monogenic obesity is rare and often causes early-onset and severe obesity. The genetic causes of syndromic monogenic obesity are not fully elucidated to date, while all genes that contribute to non-syndromic monogenic obesity are involved in energy homeostasis through the leptin-melanocortin pathway [113].

Polygenic obesity is the most common form of obesity. Meta-analysis of large-scale genome-wide association studies (GWAS) identified 941 near-independent BMI-associated single nucleotide polymorphisms (SNPs) [113], most of which were within 138 differentially expressed genes involved in neurogenesis and development of the central nervous system [114]. Loci associated with obesity overlapped with genes involved in appetite and energy regulation, lipid metabolism and adipogenesis, as well as insulin secretion and action [112]. Moreover, both familial and twin studies showed that total body fat mass as well as regional fat mass (e.g. trunk fat and lower body fat) are heritable [115].

A recent GWAS revealed SNPs in/near 25, 6 and 26 genes known to be independently associated with arm, leg and trunk fat ratios (calculated by dividing corresponding depot fat mass by total body fat mass), respectively, with another 36 genes being associated with both leg and trunk fat ratios, 3 genes associated with both arm and trunk fat ratios, and 1 gene associated with both arm and leg fat ratios [116]. Only one locus near the gene a disintegrin and metalloproteinase with thrombospondin motifs-like 3 (*ADAMTSL3*) was associated with fat ratios in all three depots [116]. *ADAMTSL3* was previously found to be associated with waist and hip circumferences [117]. Moreover, whole-exome sequencing revealed one mutation in *ADAMTSL3* that is associated with type II diabetes in a Japanese family [118]. It was also found to be differentially expressed in hepatic insulin resistant subjects [119]. Mutations in *ADAMTSL3* has also been associated with plasma HDL level alteration in families with premature coronary artery disease and acute myocardial infarction [120]. These studies suggest that SNPs in *ADAMTSL3* contribute to abnormal lipid and glucose metabolism in obesity. Sex-stratified analyses further revealed higher SNP heritability of all traits in females (~ 21–25%) than in males (~ 11–15%), with the majority of variants being associated with leg and/or trunk fat ratios [116]. SNPs of genes related to ECM maintenance and remodeling as well as cell-ECM interaction enriched in reproductive, musculoskeletal and adipose tissues were highly associated with leg and trunk fat ratios in females, but not males, indicating a critical role of ECM regulatory genes in the variability of female body fat distribution [116]. ECM maintenance is essential for adipocyte function and insulin secretion. ECM components like fibronectin, membrane type 1-matrix metalloproteinase, secreted protein acidic and rich in cysteine, collagen I and collagen VI regulate adipocyte differentiation, maturation and hypertrophy [121]. In obese humans, expression of collagen VI, α3 is decreased in sWAT, which can be reversed by weight loss, whereas leptin dose-dependently reduced its expression in vitro [122], indicating a protective role of leptin to prevent fibrosis. Mutation of the α3 chain of collagen V in ECM resulted in reduced adipocyte GLUT4 translocation and eventually insulin resistance in mice [121]. In turn, insulin also regulates the maturation of ECM components through post-transcriptional processing mediated by protein modifying and processing enzymes [123].

One study using genetic risk score, which aggregates multiple SNPs as they are inherited together from the parents, suggested that genes involved in sex hormone as well as SHBG production were also associated with vWAT accumulation [9]. Additionally, differences in sex hormone production and sex-specific body fat distribution patterns are also associated with the number of X chromosomes, as males with an extra X chromosome (e.g. Klinefelter syndrome) had less testosterone production and higher abdominal adiposity, which may be explained by the variants of genes inducing the escape of X inactivation [9].

In summary, genetic factors may predispose a person to obesity by altering central and peripheral regulation of energy homeostasis. Moreover, heritability also provides explanation for regional fat mass differences among individuals. Such heritability is more significant in females than males, which is associated with sex hormone and X chromosome activity.

### Epigenetic modifications in obesity and effects on adipose tissue metabolism

Epigenetic changes are modifications such as methylation, acetylation, phosphorylation, ubiquitination, and SUMOylation that are attached to either the DNA or histone proteins [124]. These changes are important gene expression regulatory mechanisms and can be heritable. One of the first large scale epigenome-wide association studies (EWAS) in adults from European origin revealed that methylation in intron 1 of hypoxia inducible factor 3 alpha subunit gene (*HIF3A*), which regulates cellular oxygen concentration and promotes adipocyte differentiation, was positively correlated with BMI in both whole blood and sWAT [125]. Further investigation revealed that methylation of the same locus in *HIF3A* was higher in vWAT than that in sWAT, which corresponded to lower mRNA expression in vWAT relative to sWAT in the middle-aged Caucasian population. *HIF3A* methylation in vWAT was positively correlated with sWAT, vWAT, and hip fat masses and negatively correlated with adiponectin levels after adjusting for age, gender and BMI, while that in sWAT was negatively correlated with age [126]. Moreover, greater methylation of *HIF3A* at the same position in blood DNA was also observed in obese children [127], whereas modulation of *HIF3A* methylation status by nutrients suggested that methylation is the consequence, but not cause of obesity [128]. Consistent with this conclusion, although EWAS identified over 370 CpG sites being associated with BMI, thus far, Mendelian randomization and longitudinal studies have provided evidence that changes in BMI preceded the changes in DNA methylation, and the strength of BMI-methylation association varied among ethnic groups [129]. As reviewed in [112], EWAS also showed that differentially methylated genes involved in adipogenesis, lipogenesis, fatty acid oxidation, lipoprotein metabolism as well as insulin signaling and inflammation in sWAT and/or vWAT were related to obesity traits, and about 30% of the methylated loci were further associated with the nearby SNPs, which may suggest a role for SNP-directed DNA methylation and sequential gene expression changes in obesity. A recent methylome-wide association study demonstrated that males and females with abdominal obesity shared over 75% similarities in methylation patterns in white blood cell DNA [130]. Candidate gene methylation analyses support the results from EWAS in that methylation in genes involved in appetite regulation and lipid metabolism in adipose tissue was associated with body fat mass and fat distribution, plasma triglyceride and lipoprotein levels, and genes involved in circadian rhythm regulation were also associated with obesity-related traits [112]. It should be noted that most of the EWAS recruited middle-aged Caucasian males and females although epigenetic status changes upon aging and epigenetic patterns and the strength of the association with obesity-related traits differs among ethnic groups [129]. Epigenetic status is highly influenced by environmental factors such as diet, physical activities, and medication, and methylation of the same gene in different tissues or depots may also differ [112]. Therefore, future research should focus on a wider range of ages in different ethnic groups with the comparison of methylation status in various tissues/adipose depots.

PPARγ is the master transcriptional regulator that is indispensable for adipogenesis. Decreased adipose tissue expression of *PPARγ* in obesity is associated with inflammation, lipodystrophy as well as insulin resistance [131]. Methylation of *PPARγ* in sWAT is positively correlated with vWAT mass [132], and *PPARγ* expression is lower in vWAT than sWAT [133]. Pax transactivation domain-interacting protein promotes the trimethylation of histone 3 lysine 4 (H3K4) and the enrichment of H3K4 methyltransferases in the promoter region to activate *PPARγ* [134]*.* Acetylation of H3K9 and H3K27 in the promoter region of *PPARγ* increases during adipogenesis, together with downregulation of deacetylases, which inhibits adipogenesis [135]. Activated *PPARγ* upregulates the expression of SET domain bifurcated 8—a histone lysine methyl transferase that monomethylates H4K20, which in turn further enhances the expression of *PPARγ*, as well as its downstream targets in adipogenesis [135].

Trimethylated H3K4 was also enriched in the promoter region of *LPL* and *IL-6* in normoglycemic morbid obese subjects (BMI > 40 and glucose < 100 mg/dL), and positively correlated with BMI and insulin resistance [136]. Also, there was reduced histone deacetylase 6 in the abdominal vWAT of obese subjects, which was associated with impaired deacetylation of cell death-inducing DNA fragmentation factor alpha-like effector C, leading to the fusion of lipid droplets and thus the accumulation of fat in the obese state [137].

### Stress-induced epigenetic changes in energy metabolism

Environmental factors, such as mental or nutritional stressors or pollutants, can contribute to the onset of obesity through epigenetic modifications. Maternal undernutrition during gestation causes hypermethylation of *LEP* and increased risk of dyslipidemia in the offspring [138]. Similarly, maternal overnutrition induces epigenetic changes in the offspring. Offspring from rats fed a HFD throughout gestation and lactation had higher body weights, increased sWAT and vWAT masses, and hyperleptinemia at weaning compared to those from low fat diet (LFD) fed dams [139]. Moreover, the greater body weight, epididymal fat mass as well as the higher calorie intake persisted into adulthood, even when they were switched to a LFD, compared to their LFD counterparts whose dams also consumed the LFD [139]. At the molecular level, mRNA abundance of *Npy* and *Agrp* decreased, while leptin receptor expression increased in the ARC of the offspring from HFD-fed dams, which was accompanied by hypermethylation of the promoter and enhancer regions of hypothalamic *Pomc* at weaning. While the expression of *Npy* and *Agrp* became comparable in LFD-fed offspring from either HFD- or LFD-fed dams, greater leptin receptor expression and hypermethylation in the promoter, but not enhancer of *Pomc* persisted in LFD-fed offspring from HFD-fed dams at 17 weeks after weaning. No methylation difference was detected in offspring fed either the LFD or HFD that were from LFD-fed dams, indicating the critical role of long-term maternal overnutrition in epigenetic alterations associated with appetite regulation and adipose tissue physiology in the offspring [139]. Indeed, in term placenta from obese women there was lower expression of leptin receptor and adiponectin receptors, DNA hypermethylation of *LEP* on the fetal side, as well as hypo- and hypermethylation of adiponectin and adiponectin receptor 2, respectively, on the maternal side, compared to the age-matched healthy women [140]. Changes in the enrichment of dimethylated H3K9—a transcriptional repressor in the promoter of adiponectin and monomethylated H4K20—a transcriptional activator in the promoter of leptin also contributed to maternal obesity-induced alterations in leptin and adiponectin expression in the offspring, which was associated with abnormalities in adipose tissue metabolism [141].

Maternal mental stress is also involved in the pathogenesis of obesity in the offspring through epigenetic modifications. Hypermethylation of glucocorticoid receptor gene *NR3C1* and *11β-HSD2* was observed in the cord blood of infants exposed to maternal mental stress [142]. 11β-HSD2 inhibits the conversion of cortisone to its active form cortisol and is highly expressed in the placenta [143]. Hypermethylation of *11β-HSD2* and *NR3C1* could result in lowered 11β-HSD2 activity and lowered binding of cortisol, leading to elevated free cortisol in the offspring which causes alteration in the HPA axis as well as the propensity to accumulate vWAT [38]. The positive correlation of prenatal stress exposure and offspring glucocorticoids is conserved in 14 vertebrate species according to a meta-analysis [144]. However, there are conflicting results for the effect of maternal stress exposure on offspring cortisol in human studies because cortisol/glucocorticoid levels can be affected by sample type (hair, saliva or urine), sampling time (day or night) and the age of the offspring (at birth, childhood, adolescence or adulthood), etc. [145]. Thus, one should exercise caution in drawing too many conclusions from these studies.

Long-term stress-induced chronic elevation of glucocorticoids results in over-production of leptin from adipose tissue and insulin from the pancreas, thereby reducing the sensitivity of leptin and insulin in the brain, which sequentially leads to leptin and insulin resistance and loss of appetite suppression by inhibiting NPY/AgRP neurons in the ARC [146]. Chronic stress also enhances the preference for sweet and fatty foods, which is attributed to the alteration of the mesolimbic dopamine reward system due to consistently elevated glucocorticoids [147]. Frequent ingestion of highly palatable foods repeatedly stimulates the reward system and in turn reduces the dopaminergic pathways in the brain, leading to a higher threshold of comfort feelings from palatable food, which results in overeating and obesity [147]. The gene promoters for tyrosine hydroxylase and the dopamine transporter, which are involved in dopamine synthesis and transport, respectively, were highly methylated in the ventral tegmental area (VTA)—the central reward circuitry, while those in the hypothalamus were hypomethylated compared to rats without HFD-induced obesity. These methylation differences were negatively correlated with mRNA quantities in the brain and corresponded to increased food intake but a blunted response to highly palatable food [148]. Chronic HFD ingestion also decreased the expression of μ-opioid receptor in the VTA of adult male mice, which was associated with increased DNA and H3K9 methylation, increased binding to methyl CpG-binding protein 2 and decreased acetylation of H3 in the promoter region [149]. Additionally, HFD induced hypermethylation at the promoter region of *Lep* and *Pparg2* (encoding PPARγ isoform 2 in mice) in the vWAT, but not sWAT, in adult male mice, whereas vWAT mRNA and circulating leptin were increased and *Pparg2* expression was decreased in vWAT [150]. Increased global methylation in T cells, B cells and T-cytotoxic cells were also observed in the peripheral blood of HFD-fed pigs, and was associated with the increased infiltration of macrophages in vWAT and the maintenance of inflammation in adipose tissue [151]. In summary, these results suggest that abnormalities in appetite regulation induced by chronic stress exposure during adulthood promotes excess ingestion of highly palatable food and increases adiposity through epigenetic modification of genes encoding factors that are involved in lipid metabolism and the immune response, which together contribute to the onset of metabolic diseases.

## Conclusions and implications for future research

In summary, reduced lipid mobilization and exacerbated inflammation within adipose tissue in the obese state is mainly attributed to the insensitivity/resistance of hormones driven by obesity-induced overproduction. Traits associated with appetite regulation, lipid metabolism and inflammation are heritable in obesity. Physiological changes may precede the epigenetic modifications observed in obese patients, whereas exposure to stressors during early- and later-life could promote the pathogenesis of obesity through epigenetic modifications directly in adipose tissue or through central (hypothalamic) regulation of the stress response and feeding behavior.

Epigenetic modifications are sensitive to the type and intensity of the stressors. While experimental animals can be confined to the same housing and nutritional regimen, the human epigenome is affected by diverse factors in the environment [152]. It is important to bear in mind that epigenetic modifications may not necessarily cause changes in gene expression. Thus, future research should integrate the results of epigenetic changes and gene/protein expression changes, and the analyses should incorporate environmental variables. Moreover, although studies have been carried out with rodents, focusing on the effect of maternal overnutrition and distress on metabolic alterations in the offspring, there is little knowledge at the molecular level, especially the direct epigenetic changes in adipose tissue. It is of vital importance to understand the role of epigenetic modifications in stress-induced alterations in adipose tissue physiology and corresponding mechanisms. Such information is critical to facilitate the identification of novel preventative strategies and therapeutic targets to manage stress-induced obesity and metabolic diseases.

## Data Availability

Not applicable.

## Abbreviations

11β-HSD: 11β-Hydroxysteroid dehydrogenase
12,13-diHOME: 12,13-Dihydroxy-9Z-octadecenoic acid
ACTH: Adrenocorticotropic hormone
ADAMTSL3: A disintegrin and metalloproteinase with thrombospondin motifs-like 3
AgRP: Agouti-related peptide
ARC: Arcuate nucleus
BAT: Brown adipose tissue
BBB: Blood brain barrier
BMI: Body mass index
CART: Cocaine-amphetamine-regulated transcript
CpG: 5′-Cytosine-phosphate-guanine-3’
CRF: Corticotropin-releasing factor
ECM: Extracellular matrix
EWAS: Epigenome-wide association study
FAHFA: Fatty acid ester of hydroxyl fatty acid
FFA: Free fatty acid
FSH: Follicle-stimulating hormone
FTO: Fat mass and obesity associated gene
GH: Growth hormone
GHS-R: Growth hormone secretagogue receptor
GLUT4: Glucose transporter type 4
GnRH: Gonadotropin-releasing hormone
GR: Glucocorticoid receptor
GWAS: Genome-wide association study
H3K4: Histone 3 lysine 4
HDL: High density lipoprotein
HFD: High fat diet
HIF3A: Hypoxia inducible factor 3 alpha
HOX: Homeobox
HPA: Hypothalamus–pituitary–adrenal
iMAT: Intramuscular adipose tissue
itMAT: Intermuscular adipose tissue
IGF-1: Insulin-like growth factor 1
LEP: Leptin
LFD: Low fat diet
LH: Luteinizing hormone
LPL: Lipoprotein lipase
MAT: Marrow adipose tissue
MR: Mineralocorticoid receptors
NPY: Neuropeptide Y
NR3C1: Nuclear receptor subfamily 3 group C member 1
OX1R: Orexin receptor 1
POMC: Proopiomelanocortin
PPARγ: Peroxisome proliferator-activated receptor gamma
PVN: Paraventricular nucleus
SHBG: Sex hormone binding globulin
SNP: Single nucleotide polymorphism
sWAT: Subcutaneous adipose tissue
T3: Triiodothyronine
TSH: Thyroid-stimulating hormone
UCP: Uncoupling protein
VTA: Ventral tegmental area
vWAT: Visceral white adipose tissue
WAT: White adipose tissue

## References

[1] Galic S, Oakhill JS, Steinberg GR (2010). Adipose tissue as an endocrine organ. Mol Cell Endocrinol.

[2] Kershaw EE, Flier JS (2004). Adipose tissue as an endocrine organ. J Clin Endocrinol Metab.

[3] Trayhurn P, Beattie JH (2001). Physiological role of adipose tissue: white adipose tissue as an endocrine and secretory organ. Proc Nutr Soc.

[4] Berry DC, Stenesen D, Zeve D, Graff JM (2013). The developmental origins of adipose tissue. Development (Cambridge, England).

[5] Mulya A, Kirwan JP (2016). Brown and beige adipose tissue: Therapy for obesity and its comorbidities?. Endocrinol Metab Clin North Am.

[6] Hausman GJ, Basu U, Du M, Fernyhough-Culver M, Dodson MV (2014). Intermuscular and intramuscular adipose tissues: bad vs. good adipose tissues. Adipocyte.

[7] Poulos SP, Hausman DB, Hausman GJ (2010). The development and endocrine functions of adipose tissue. Mol Cell Endocrinol.

[8] Park A, Kim WK, Bae KH (2014). Distinction of white, beige and brown adipocytes derived from mesenchymal stem cells. World J Stem Cells.

[9] Frank AP, de Souza SR, Palmer BF, Clegg DJ (2019). Determinants of body fat distribution in humans may provide insight about obesity-related health risks. J Lipid Res.

[10] Bergman RN, Kim SP, Catalano KJ, Hsu IR, Chiu JD, Kabir M (2006). Why visceral fat is Bad: mechanisms of the metabolic syndrome. Obesity.

[11] Snijder MB, Dekker JM, Visser M, Bouter LM, Stehouwer CD, Yudkin JS (2004). Trunk fat and leg fat have independent and opposite associations with fasting and postload glucose levels: The Hoorn study. Diabetes Care.

[12] Ailhaud G, Hauner H, Bray AG, Bouchard C, James WPM (2003). Development of white adipose tissue. Handbook of obesity.

[13] Moreno-Navarrete JM, Fernández-Real JM (2012). Adipocyte differentiation.

[14] Therrien F, Drapeau V, Lalonde J, Lupien SJ, Beaulieu S, Tremblay A (2007). Awakening cortisol response in lean, obese, and reduced obese individuals: Effect of gender and fat distribution. Obesity (Silver Spring).

[15] Abraham SB, Rubino D, Sinaii N, Ramsey S, Nieman LK (2013). Cortisol, obesity, and the metabolic syndrome: A cross-sectional study of obese subjects and review of the literature. Obesity (Silver Spring).

[16] Rask E, Walker BR, Soderberg S, Livingstone DE, Eliasson M, Johnson O (2002). Tissue-specific changes in peripheral cortisol metabolism in obese women: Increased adipose 11beta-hydroxysteroid dehydrogenase type 1 activity. J Clin Endocrinol Metab.

[17] Long NM, Nathanielsz PW, Ford SP (2012). The impact of maternal overnutrition and obesity on hypothalamic-pituitary-adrenal axis response of offspring to stress. Domest Anim Endocrinol.

[18] Shasa DR, Odhiambo JF, Long NM, Tuersunjiang N, Nathanielsz PW, Ford SP (2015). Multigenerational impact of maternal overnutrition/obesity in the sheep on the neonatal leptin surge in granddaughters. Int J Obes (Lond).

[19] World Health Organization. Obesity and overweight. 2018. https://www.who.int/news-room/fact-sheets/detail/obesity-and-overweight

[20] Peltz G, Aguirre MT, Sanderson M, Fadden MK (2010). The role of fat mass index in determining obesity. Am J Hum Biol.

[21] Nuttall FQ (2015). Body mass index: Obesity, BMI, and health: A critical review. Nutr Today.

[22] Flegal KM, Ogden CL, Yanovski JA, Freedman DS, Shepherd JA, Graubard BI (2010). High adiposity and high body mass index-for-age in US children and adolescents overall and by race-ethnic group. Am J Clin Nutr.

[23] Flegal KM, Carroll MD, Kit BK, Ogden CL (2012). Prevalence of obesity and trends in the distribution of body mass index among US adults, 1999–2010. JAMA.

[24] Heymsfield SB, Peterson CM, Thomas DM, Heo M, Schuna JM (2016). Why are there race/ethnic differences in adult body mass index-adiposity relationships? A quantitative critical review. Obes Rev.

[25] Bosy-Westphal A, Geisler C, Onur S, Korth O, Selberg O, Schrezenmeir J (2006). Value of body fat mass vs anthropometric obesity indices in the assessment of metabolic risk factors. Int J Obes (Lond).

[26] Borrud LG, Flegal KM, Looker AC, Everhart JE, Harris TB, Shepherd JA (2010). Body composition data for individuals 8 years of age and older: US population 1999–2004. Vital Health Stat.

[27] Lear SA, Humphries KH, Kohli S, Chockalingam A, Frohlich JJ, Birmingham CL (2007). Visceral adipose tissue accumulation differs according to ethnic background: results of the Multicultural Community Health Assessment Trial (M-CHAT). Am J Clin Nutr.

[28] Cypess AM, Lehman S, Williams G, Tal I, Rodman D, Goldfine AB (2009). Identification and importance of brown adipose tissue in adult humans. N Engl J Med.

[29] Saito M, Okamatsu-Ogura Y, Matsushita M, Watanabe K, Yoneshiro T, Nio-Kobayashi J (2009). High incidence of metabolically active brown adipose tissue in healthy adult humans: effects of cold exposure and adiposity. Diabetes.

[30] Vijgen GH, Bouvy ND, Teule GJ, Brans B, Schrauwen P, van Marken Lichtenbelt WD (2011). Brown adipose tissue in morbidly obese subjects. PLoS ONE.

[31] Leitner BP, Huang S, Brychta RJ, Duckworth CJ, Baskin AS, McGehee S (2017). Mapping of human brown adipose tissue in lean and obese young men. Proc Natl Acad Sci USA.

[32] Tchkonia T, Thomou T, Zhu Y, Karagiannides I, Pothoulakis C, Jensen MD (2013). Mechanisms and metabolic implications of regional differences among fat depots. Cell Metab.

[33] Ahn J, Wu H, Lee K (2019). Integrative analysis revealing human adipose-specific genes and consolidating obesity loci. Sci Rep.

[34] Linder K, Arner P, Flores-Morales A, Tollet-Egnell P, Norstedt G (2004). Differentially expressed genes in visceral or subcutaneous adipose tissue of obese men and women. J Lipid Res.

[35] Passaro A, Miselli MA, Sanz JM, Dalla Nora E, Morieri ML, Colonna R (2017). Gene expression regional differences in human subcutaneous adipose tissue. BMC genomics.

[36] Bradford ST, Nair SS, Statham AL, van Dijk SJ, Peters TJ, Anwar F (2019). Methylome and transcriptome maps of human visceral and subcutaneous adipocytes reveal key epigenetic differences at developmental genes. Sci Rep.

[37] Jo J, Gavrilova O, Pack S, Jou W, Mullen S, Sumner AE (2009). Hypertrophy and/or hyperplasia: dynamics of adipose tissue growth. PLoS Comput Biol.

[38] Booth A, Magnuson A, Foster M (2014). Detrimental and protective fat: body fat distribution and its relation to metabolic disease. Horm Mol Biol Clin Investig.

[39] Guo Z, Hensrud DD, Johnson CM, Jensen MD (1999). Regional postprandial fatty acid metabolism in different obesity phenotypes. Diabetes.

[40] Goossens GH (2017). The metabolic phenotype in obesity: Fat mass, body fat distribution, and adipose tissue function. Obes Facts.

[41] Singhal V, Bose A, Liang Y, Srivastava G, Goode S, Stanford FC (2019). Marrow adipose tissue in adolescent girls with obesity. Bone.

[42] Li Y, Meng Y, Yu X (2019). The unique metabolic characteristics of bone aarrow adipose tissue. Front Endocrinol.

[43] Veldhuis-Vlug AG, Rosen CJ (2018). Clinical implications of bone marrow adiposity. J Intern Med.

[44] Pasarica M, Sereda OR, Redman LM, Albarado DC, Hymel DT, Roan LE (2009). Reduced adipose tissue oxygenation in human obesity: evidence for rarefaction, macrophage chemotaxis, and inflammation without an angiogenic response. Diabetes.

[45] Mattacks CA, Sadler D, Pond CM (2003). The cellular structure and lipid/protein composition of adipose tissue surrounding chronically stimulated lymph nodes in rats. J Anat.

[46] Castro AM, Macedo-de la Concha LE, Pantoja-Meléndez CA (2017). Low-grade inflammation and its relation to obesity and chronic degenerative diseases. Rev Med Hosp Gen (Mex).

[47] van den Berg SM, van Dam AD, Rensen PC, de Winther MP, Lutgens E (2017). Immune modulation of brown(ing) adipose tissue in obesity. Endocr Rev.

[48] Alcala M, Calderon-Dominguez M, Serra D, Herrero L, Viana M (2019). Mechanisms of impaired brown adipose tissue recruitment in obesity. Front Physiol.

[49] Thomou T, Mori MA, Dreyfuss JM, Konishi M, Sakaguchi M, Wolfrum C (2017). Adipose-derived circulating miRNAs regulate gene expression in other tissues. Nature.

[50] Ferrante SC, Nadler EP, Pillai DK, Hubal MJ, Wang Z, Wang JM (2015). Adipocyte-derived exosomal miRNAs: a novel mechanism for obesity-related disease. Pediatr Res.

[51] Laudes M (2011). Role of WNT signalling in the determination of human mesenchymal stem cells into preadipocytes. J Mol Endocrinol.

[52] Li S, Wu J (2020). TGF-β/SMAD signaling regulation of mesenchymal stem cells in adipocyte commitment. Stem Cell Res Ther.

[53] Zhang M, Zhou Z, Wang J, Li S (2016). MiR-130b promotes obesity associated adipose tissue inflammation and insulin resistance in diabetes mice through alleviating M2 macrophage polarization via repression of PPAR-γ. Immunol Lett.

[54] Xu F, Xiang Q, Huang J, Chen Q, Yu N, Long X (2019). Exosomal miR-423-5p mediates the proangiogenic activity of human adipose-derived stem cells by targeting Sufu. Stem Cell Res Ther.

[55] Ortega FJ, Mercader JM, Catalán V, Moreno-Navarrete JM, Pueyo N, Sabater M (2013). Targeting the circulating microRNA signature of obesity. Clin Chem.

[56] Prats-Puig A, Ortega FJ, Mercader JM, Moreno-Navarrete JM, Moreno M, Bonet N (2013). Changes in circulating microRNAs are associated with childhood obesity. J Clin Endocrinol Metab.

[57] Chen Y, Buyel JJ, Hanssen MJW, Siegel F, Pan R, Naumann J (2016). Exosomal microRNA miR-92a concentration in serum reflects human brown fat activity. Nat Commun.

[58] Parahuleva MS, Lipps C, Parviz B, Hölschermann H, Schieffer B, Schulz R (2018). MicroRNA expression profile of human advanced coronary atherosclerotic plaques. Sci Rep.

[59] Cao H, Gerhold K, Mayers JR, Wiest MM, Watkins SM, Hotamisligil GS (2008). Identification of a lipokine, a lipid hormone linking adipose tissue to systemic metabolism. Cell.

[60] Lynes MD, Leiria LO, Lundh M, Bartelt A, Shamsi F, Huang TL (2017). The cold-induced lipokine 12,13-diHOME promotes fatty acid transport into brown adipose tissue. Nat Med.

[61] Zhu Q, Yan J, Ni J, Feng Y (2020). FAHFA footprint in the visceral fat of mice across their lifespan. Biochim Biophys Acta-Mol cell Biol L.

[62] May FJ, Baer LA, Lehnig AC, So K, Chen EY, Gao F (2017). Lipidomic adaptations in white and brown adipose tissue in response to exercise demonstrate molecular species-specific remodeling. Cell Rep.

[63] Yore MM, Syed I, Moraes-Vieira PM, Zhang T, Herman MA, Homan EA (2014). Discovery of a class of endogenous mammalian lipids with anti-diabetic and anti-inflammatory effects. Cell.

[64] Halaas JL, Gajiwala KS, Maffei M, Cohen SL, Chait BT, Rabinowitz D (1995). Weight-reducing effects of the plasma protein encoded by the obese gene. Science.

[65] Sidhu S, Parikh T, Burman KD, Feingold KR, Anawalt B, Boyce A, Chrousos G, Dungan K, Grossman A (2000). Endocrine changes in obesity. Endotext.

[66] Thaler JP, Guyenet SJ, Dorfman MD, Wisse BE, Schwartz MW (2013). Hypothalamic inflammation: Marker or mechanism of obesity pathogenesis?. Diabetes.

[67] Hahn TM, Breininger JF, Baskin DG, Schwartz MW (1998). Coexpression of Agrp and NPY in fasting-activated hypothalamic neurons. Nat Neurosci.

[68] Minor RK, Chang JW, de Cabo R (2009). Hungry for life: How the arcuate nucleus and neuropeptide Y may play a critical role in mediating the benefits of calorie restriction. Mol Cell Endocrinol.

[69] Amitani M, Asakawa A, Amitani H, Inui A (2013). The role of leptin in the control of insulin-glucose axis. Front Neurosci.

[70] Carter S, Caron A, Richard D, Picard F (2013). Role of leptin resistance in the development of obesity in older patients. Clin Interv Aging.

[71] Di Spiezio A, Sandin ES, Dore R, Muller-Fielitz H, Storck SE, Bernau M (2018). The LepR-mediated leptin transport across brain barriers controls food reward. Mol Metab.

[72] Sainz N, Barrenetxe J, Moreno-Aliaga MJ, Martinez JA (2015). Leptin resistance and diet-induced obesity: Central and peripheral actions of leptin. Metabolism.

[73] Anubhuti AS (2008). Leptin and its metabolic interactions: an update. Diabetes Obes Metab.

[74] Tschop M, Weyer C, Tataranni PA, Devanarayan V, Ravussin E, Heiman ML (2001). Circulating ghrelin levels are decreased in human obesity. Diabetes.

[75] Andrews ZB, Liu ZW, Walllingford N, Erion DM, Borok E, Friedman JM (2008). UCP2 mediates ghrelin's action on NPY/AgRP neurons by lowering free radicals. Nature.

[76] Mahadik SR, Lele RD, Saranath D, Seth A, Parikh V (2012). Uncoupling protein-2 (UCP2) gene expression in subcutaneous and omental adipose tissue of Asian Indians: relationship to adiponectin and parameters of metabolic syndrome. Adipocyte.

[77] Zhang CY, Baffy G, Perret P, Krauss S, Peroni O, Grujic D (2001). Uncoupling protein-2 negatively regulates insulin secretion and is a major link between obesity, beta cell dysfunction, and type 2 diabetes. Cell.

[78] Linnemann AK, Baan M, Davis DB (2014). Pancreatic β-cell proliferation in obesity. Adv Nutr.

[79] Golson ML, Misfeldt AA, Kopsombut UG, Petersen CP, Gannon M (2010). High fat diet regulation of β-cell proliferation and β-cell mass. Open Endocrinol J..

[80] Meier U, Gressner AM (2004). Endocrine regulation of energy metabolism: Review of pathobiochemical and clinical chemical aspects of leptin, ghrelin, adiponectin, and resistin. Clin Chem.

[81] Douyon L, Schteingart DE (2002). Effect of obesity and starvation on thyroid hormone, growth hormone, and cortisol secretion. Endocrinol Metab Clin North Am.

[82] Farkas I, Vastagh C, Sárvári M, Liposits Z (2013). Ghrelin decreases firing activity of gonadotropin-releasing hormone (GnRH) neurons in an estrous cycle and endocannabinoid signaling dependent manner. PLoS ONE.

[83] Smith JT, Acohido BV, Clifton DK, Steiner RA (2006). KiSS-1 neurones are direct targets for leptin in the ob/ob mouse. J Neuroendocrinol.

[84] Harter CJL, Kavanagh GS, Smith JT (2018). The role of kisspeptin neurons in reproduction and metabolism. J Endocrinol.

[85] Quennell JH, Howell CS, Roa J, Augustine RA, Grattan DR, Anderson GM (2011). Leptin deficiency and diet-induced obesity reduce hypothalamic kisspeptin expression in mice. Endocrinology.

[86] Zhai L, Zhao J, Zhu Y, Liu Q, Niu W, Liu C (2018). Downregulation of leptin receptor and kisspeptin/GPR54 in the murine hypothalamus contributes to male hypogonadism caused by high-fat diet-induced obesity. Endocrine.

[87] De Pergola G, Maldera S, Tartagni M, Pannacciulli N, Loverro G, Giorgino R (2006). Inhibitory effect of obesity on gonadotropin, estradiol, and inhibin B levels in fertile women. Obesity (Silver Spring).

[88] Beydoun HA, Beydoun MA, Wiggins N, Stadtmauer L (2012). Relationship of obesity-related disturbances with LH/FSH ratio among post-menopausal women in the United States. Maturitas.

[89] Bekaert M, Van Nieuwenhove Y, Calders P, Cuvelier CA, Batens A-H, Kaufman J-M (2015). Determinants of testosterone levels in human male obesity. Endocrine.

[90] Freeman EW, Sammel MD, Lin H, Gracia CR (2010). Obesity and reproductive hormone levels in the transition to menopause. Menopause.

[91] Stanikova D, Zsido RG, Luck T, Pabst A, Enzenbach C, Bae YJ (2019). Testosterone imbalance may link depression and increased body weight in premenopausal women. Transl Psychiatry.

[92] Poddar M, Chetty Y, Chetty VT (2017). How does obesity affect the endocrine system? A narrative review. Clin Obes.

[93] Fui MNT, Dupuis P, Grossmann M (2014). Lowered testosterone in male obesity: Mechanisms, morbidity and management. Asian J Androl.

[94] Buster JE. Estrogen kinetics for clinicians2008. https://www.glowm.com/section_view/heading/Estrogen%20Kinetics%20for%20Clinicians/item/279.

[95] Pedersen SB, Kristensen K, Hermann PA, Katzenellenbogen JA, Richelsen B (2004). Estrogen controls lipolysis by up-regulating alpha2A-adrenergic receptors directly in human adipose tissue through the estrogen receptor alpha. Implications for the female fat distribution. J Clin Endocrinol Metab..

[96] Chang E, Varghese M, Singer K (2018). Gender and sex differences in adipose tissue. Curr Diab Rep.

[97] Hirschberg AL (2012). Sex hormones, appetite and eating behaviour in women. Maturitas.

[98] Suemaru S, Hashimoto K, Hattori T, Inoue H, Kageyama J, Ota Z (1986). Starvation-induced changes in rat brain corticotropin-releasing factor (CRF) and pituitary-adrenocortical response. Life Sci.

[99] Yang K, Guan H, Arany E, Hill DJ, Cao X (2008). Neuropeptide Y is produced in visceral adipose tissue and promotes proliferation of adipocyte precursor cells via the Y1 receptor. FASEB J.

[100] Long M, Zhou J, Li D, Zheng L, Xu Z, Zhou S (2015). Long-term over-expression of neuropeptide Y in hypothalamic paraventricular nucleus contributes to adipose tissue insulin resistance partly via the Y5 receptor. PLoS ONE.

[101] Sakamoto F, Yamada S, Ueta Y (2004). Centrally administered orexin-A activates corticotropin-releasing factor-containing neurons in the hypothalamic paraventricular nucleus and central amygdaloid nucleus of rats: possible involvement of central orexins on stress-activated central CRF neurons. Regul Pept.

[102] Ziolkowska A, Spinazzi R, Albertin G, Nowak M, Malendowicz LK, Tortorella C (2005). Orexins stimulate glucocorticoid secretion from cultured rat and human adrenocortical cells, exclusively acting via the OX1 receptor. J Steroid Biochem Mol Biol.

[103] Winsky-Sommerer R, Yamanaka A, Diano S, Borok E, Roberts AJ, Sakurai T (2004). Interaction between the corticotropin-releasing factor system and hypocretins (orexins): a novel circuit mediating stress response. J Neurosci.

[104] Dube MG, Kalra SP, Kalra PS (1999). Food intake elicited by central administration of orexins/hypocretins: Identification of hypothalamic sites of action. Brain Res.

[105] Shen Y, Zhao Y, Zheng D, Chang X, Ju S, Guo L (2013). Effects of orexin A on GLUT4 expression and lipid content via MAPK signaling in 3T3-L1 adipocytes. J Steroid Biochem Mol Biol.

[106] Park JH, Shim HM, Na AY, Bae JH, Im SS, Song DK (2015). Orexin A regulates plasma insulin and leptin levels in a time-dependent manner following a glucose load in mice. Diabetologia.

[107] Kluge M, Riedl S, Uhr M, Schmidt D, Zhang X, Yassouridis A (2010). Ghrelin affects the hypothalamus-pituitary-thyroid axis in humans by increasing free thyroxine and decreasing TSH in plasma. Eur J Endocrinol.

[108] Iacobellis G, Ribaudo MC, Zappaterreno A, Iannucci CV, Leonetti F (2005). Relationship of thyroid function with body mass index, leptin, insulin sensitivity and adiponectin in euthyroid obese women. Clin Endocrinol (Oxf).

[109] Biondi B (2010). Thyroid and obesity: An intriguing relationship. J Clin Endocrinol Metab.

[110] Pearce EN (2012). Thyroid hormone and obesity. Curr Opin Endocrinol Diabetes Obes.

[111] Reinehr T (2010). Obesity and thyroid function. Mol Cell Endocrinol.

[112] Rohde K, Keller M, la Cour PL, Bluher M, Kovacs P, Bottcher Y (2019). Genetics and epigenetics in obesity. Metabolism.

[113] Pigeyre M, Yazdi FT, Kaur Y, Meyre D (2016). Recent progress in genetics, epigenetics and metagenomics unveils the pathophysiology of human obesity. Clin Sci (Lond).

[114] Yengo L, Sidorenko J, Kemper KE, Zheng Z, Wood AR, Weedon MN (2018). Meta-analysis of genome-wide association studies for height and body mass index in approximately 700000 individuals of European ancestry. Hum Mol Genet.

[115] Schleinitz D, Bottcher Y, Bluher M, Kovacs P (2014). The genetics of fat distribution. Diabetologia.

[116] Rask-Andersen M, Karlsson T, Ek WE, Johansson Å (2019). Genome-wide association study of body fat distribution identifies adiposity loci and sex-specific genetic effects. Nat Commun.

[117] Zillikens MC, Demissie S, Hsu YH, Yerges-Armstrong LM, Chou WC, Stolk L (2017). Large meta-analysis of genome-wide association studies identifies five loci for lean body mass. Nat Commun.

[118] Jambaljav B, Tanaka D, Nagashima K, Harashima SI, Harada N, Harada T (2018). Whole-exome sequencing in a Japanese family with highly aggregated diabetes identifies a candidate susceptibility mutation in ADAMTSL3. Diabetes Res Clin Pract.

[119] van der Kolk BW, Kalafati M, Adriaens M, van Greevenbroek MMJ, Vogelzangs N, Saris WHM (2019). Subcutaneous adipose tissue and systemic inflammation are associated with peripheral but not hepatic insulin resistance in humans. Diabetes.

[120] Yang R, Li L, Seidelmann SB, Shen G-Q, Sharma S, Rao S (2010). A genome-wide linkage scan identifies multiple quantitative trait loci for HDL-cholesterol levels in families with premature CAD and MI. J Lipid Res.

[121] Huang G, Greenspan DS (2012). ECM roles in the function of metabolic tissues. Trends Endocrinol Metab.

[122] McCulloch LJ, Rawling TJ, Sjöholm K, Franck N, Dankel SN, Price EJ (2015). COL6A3 is regulated by leptin in human adipose tissue and reduced in obesity. Endocrinology.

[123] Wang P, Keijer J, Bunschoten A, Bouwman F, Renes J, Mariman E (2006). Insulin modulates the secretion of proteins from mature 3T3-L1 adipocytes: A role for transcriptional regulation of processing. Diabetologia.

[124] Weinhold B (2006). Epigenetics: The science of change. Environ Health Perspect.

[125] Dick KJ, Nelson CP, Tsaprouni L, Sandling JK, Aissi D, Wahl S (2014). DNA methylation and body-mass index: a genome-wide analysis. Lancet.

[126] Pfeiffer S, Krüger J, Maierhofer A, Böttcher Y, Klöting N, El Hajj N (2016). Hypoxia-inducible factor 3A gene expression and methylation in adipose tissue is related to adipose tissue dysfunction. Sci Rep.

[127] Wang S, Song J, Yang Y, Zhang Y, Wang H, Ma J (2015). HIF3A DNA methylation is associated with childhood obesity and ALT. PLoS ONE.

[128] Bell CG (2017). The epigenomic analysis of human obesity. Obesity (Silver Spring).

[129] Sun D, Zhang T, Su S, Hao G, Chen T, Li QZ (2019). Body mass index drives changes in DNA methylation: a longitudinal study. Circ Res.

[130] Arpón A, Milagro FI, Ramos-Lopez O, Mansego ML, Riezu-Boj J-I, Martínez JA (2019). Methylome-wide association study in peripheral white blood cells focusing on central obesity and inflammation. Genes (Basel).

[131] Corrales P, Vidal-Puig A, Medina-Gomez G (2018). PPARs and metabolic disorders associated with challenged adipose tissue plasticity. Int J Mol Sci.

[132] Drogan D, Boeing H, Janke J, Schmitt B, Zhou Y, Walter J (2015). Regional distribution of body fat in relation to DNA methylation within the LPL, ADIPOQ and PPARγ promoters in subcutaneous adipose tissue. Nutr Diabetes.

[133] Garin-Shkolnik T, Rudich A, Hotamisligil GS, Rubinstein M (2014). FABP4 attenuates PPARgamma and adipogenesis and is inversely correlated with PPARgamma in adipose tissues. Diabetes.

[134] Cho Y-W, Hong S, Jin Q, Wang L, Lee J-E, Gavrilova O (2009). Histone methylation regulator PTIP is required for PPARgamma and C/EBPalpha expression and adipogenesis. Cell Metab.

[135] Huang Q, Ma C, Chen L, Luo D, Chen R, Liang F (2018). Mechanistic insights into the interaction between transcription factors and epigenetic modifications and the contribution to the development of obesity. Front Endocrinol (Lausanne).

[136] Castellano-Castillo D, Denechaud PD, Fajas L, Moreno-Indias I, Oliva-Olivera W, Tinahones F (2019). Human adipose tissue H3K4me3 histone mark in adipogenic, lipid metabolism and inflammatory genes is positively associated with BMI and HOMA-IR. PLoS ONE.

[137] Qian H, Chen Y, Nian Z, Su L, Yu H, Chen FJ (2017). HDAC6-mediated acetylation of lipid droplet-binding protein CIDEC regulates fat-induced lipid storage. J Clin Invest.

[138] Tobi EW, Lumey LH, Talens RP, Kremer D, Putter H, Stein AD (2009). DNA methylation differences after exposure to prenatal famine are common and timing- and sex-specific. Hum Mol Genet.

[139] Gali Ramamoorthy T, Allen TJ, Davies A, Harno E, Sefton C, Murgatroyd C (2018). Maternal overnutrition programs epigenetic changes in the regulatory regions of hypothalamic Pomc in the offspring of rats. Int J Obes (Lond).

[140] Nogues P, Dos Santos E, Jammes H, Berveiller P, Arnould L, Vialard F (2019). Maternal obesity influences expression and DNA methylation of the adiponectin and leptin systems in human third-trimester placenta. Clin Epigenetics.

[141] Masuyama H, Mitsui T, Nobumoto E, Hiramatsu Y (2015). The effects of high-fat diet exposure in utero on the obesogenic and diabetogenic traits through epigenetic changes in adiponectin and leptin gene expression for multiple generations in female mice. Endocrinology.

[142] Jensen Peña C, Monk C, Champagne FA (2012). Epigenetic effects of prenatal stress on 11β-hydroxysteroid dehydrogenase-2 in the placenta and fetal brain. PLoS ONE.

[143] Seth S, Lewis AJ, Saffery R, Lappas M, Galbally M (2015). Maternal prenatal mental health and placental 11β-HSD2 gene expression: Initial findings from the mercy pregnancy and emotional wellbeing study. Int J Mol Sci.

[144] Thayer ZM, Wilson MA, Kim AW, Jaeggi AV (2018). Impact of prenatal stress on offspring glucocorticoid levels: A phylogenetic meta-analysis across 14 vertebrate species. Sci Rep.

[145] Send TS, Bardtke S, Gilles M, Wolf IAC, Sutterlin MW, Wudy SA (2019). Prenatal maternal stress is associated with lower cortisol and cortisone levels in the first morning urine of 45-month-old children. Psychoneuroendocrinology.

[146] Sominsky L, Spencer SJ (2014). Eating behavior and stress: A pathway to obesity. Front Psychol.

[147] Adam TC, Epel ES (2007). Stress, eating and the reward system. Physiol Behav.

[148] Vucetic Z, Carlin JL, Totoki K, Reyes TM (2012). Epigenetic dysregulation of the dopamine system in diet-induced obesity. J Neurochem.

[149] Vucetic Z, Kimmel J, Reyes TM (2011). Chronic high-fat diet drives postnatal epigenetic regulation of mu-opioid receptor in the brain. Neuropsychopharmacology.

[150] Zwamborn RAJ, Slieker RC, Mulder PCA, Zoetemelk I, Verschuren L, Suchiman HED (2017). Prolonged high-fat diet induces gradual and fat depot-specific DNA methylation changes in adult mice. Sci Rep.

[151] Jacobsen MJ, Mentzel CMJ, Olesen AS, Huby T, Jørgensen CB, Barrès R (2016). Altered methylation profile of lymphocytes is concordant with perturbation of lipids metabolism and inflammatory response in obesity. J Diabetes Res.

[152] Shen L, Li C, Wang Z, Zhang R, Shen Y, Miles T (2019). Early-life exposure to severe famine is associated with higher methylation level in the IGF2 gene and higher total cholesterol in late adulthood: the Genomic Research of the Chinese Famine (GRECF) study. Clin Epigenet.

